# Paramedic Initiated Lisinopril For Acute Stroke Treatment (PIL-FAST): study protocol for a pilot randomised controlled trial

**DOI:** 10.1186/1745-6215-12-152

**Published:** 2011-06-15

**Authors:** Lisa Shaw, Christopher Price, Sally McLure, Denise Howel, Elaine McColl, Gary A Ford

**Affiliations:** 1 Institute for Ageing and Health (Stroke Research Group), Newcastle University, Newcastle upon Tyne, NE4 5PL, England, UK; 2 Northumbria Healthcare NHS Foundation Trust, Wansbeck General Hospital, Ashington, Northumberland, NE63 9JJ, England, UK; 3 North East Ambulance Service NHS Trust, Bernicia House, Newburn Riverside, Newcastle upon Tyne, NE15 8NY, England, UK; 4 Institute of Health and Society, Newcastle University, Newcastle upon Tyne, NE2 4AX, England, UK; 5 Newcastle upon Tyne Hospitals NHS Foundation Trust, Level 6, Leazes Wing, Royal Victoria Infirmary, Newcastle upon Tyne, NE1 4LP, England, UK

## Abstract

**Background:**

High blood pressure during acute stroke is associated with poorer stroke outcome. Previous trials have failed to show benefit from lowering blood pressure but treatment may have been commenced too late to be effective. The earliest that acute stroke treatments could be initiated is during contact with the emergency medical services (paramedics). However, experience of pre-hospital clinical trials is limited and logistical challenges are likely to be greater than for trials performed in other settings. We report the protocol for a pilot randomised controlled trial of paramedic initiated blood pressure lowering treatment for hypertension in acute stroke.

**Methods:**

Trial Design: Double blind parallel group external pilot randomised controlled trial.

Setting: Participant recruitment and initial treatment by North East Ambulance Service research trained paramedics responding to the emergency call. Continued treatment in three study hospitals.

Participants: Target is recruitment of 60 adults with acute arm weakness due to suspected stroke (within 3 hours of symptom onset) and hypertension (systolic BP>160 mmHg).

Intervention: Lisinopril 5-10 mg (intervention group), matched placebo (control group), daily for 7 days.

Randomisation: Study medication contained within identical pre-randomised "trial packs" carried by research trained paramedics.

Outcomes: Study feasibility (recruitment rate, compliance with data collection) and clinical data to inform the design of a definitive randomised controlled trial (blood pressure monitoring, National Institute of Health Stroke Scale, Barthel ADL Index, Modified Rankin Scale, renal function).

**Discussion:**

This pilot study is assessing the feasibility of a randomised controlled trial of paramedic initiated lisinopril for hypertension early after the onset of acute stroke. The results will inform the design of a definitive RCT to evaluate the effects of very early blood pressure lowering in acute stroke.

**Trial Registration:**

EudraCT: 2010-019180-10

ClinicalTrials.gov: NCT01066572

ISRCTN: 54540667

## Background

Systemic hypertension occurs commonly during acute stroke and is associated with increased neurological impairment, poor functional outcome and death [1,2]. Due to the simultaneous disruption of cerebral vascular autoregulation that occurs in acute stroke, systemic hypertension has a greater effect on cerebral blood flow than normal [3]. This can lead to excessive cerebral oedema during ischaemic stroke or haematoma expansion after haemorrhage, both of which are associated with greater neurological injury [4]. Low levels of blood pressure may also be harmful, however, probably because of cerebral hypoperfusion [5]. Observational data supports a U-shaped relation between baseline systolic blood pressure and outcome. For every 10 mmHg rise above 150 mmHg the risk of early death rises by 3.8% but for every 10 mmHg fall below 150 mmHg the risk of early death rises by 17.9% [5].

Optimal treatment of hypertension in acute stroke is unclear. A Cochrane meta-analysis of interventions for deliberately altering blood pressure in acute stroke showed that significant reductions in blood pressure readings were possible, but functional outcome and death were not altered by any of the drugs used [6]. More recent acute blood pressure lowering trials have also shown no benefit on survival or reduction in disability [7]. The explanations for this include that interventions to lower blood pressure do not influence mechanisms of progressive cerebral injury, and that trials have not been of sufficient size to detect a treatment effect [6,8]. However, a key reason may be that blood pressure lowering treatment was started too late after stroke onset to be effective. Acute stroke treatments are known to be time dependent due to the rapid speed at which neuronal injury occurs following cerebral hypoxia and oedema [9]. This has been very clearly demonstrated by the pooled data from trials assessing the effects of intravenous thrombolysis on acute ischaemic stroke where every minute of delay resulted in a reduced chance of a better outcome until 4.5 - 6 hours after symptom onset[10]. Current hospital based trials of blood pressure lowering following cerebral haemorrhage have recognised the importance of treatment timing and are being performed with time windows of 6 hours or less [11,12].

The earliest that patients suffering an acute stroke could receive treatment or be invited to participate in a clinical trial is during contact with the emergency medical services (paramedics). Such pre-hospital intervention could result in an important reduction in time to treatment which may influence stroke outcome. Although pre-hospital clinical trials are not a new concept, few have been conducted to date [13,14]. In terms of pre-hospital stroke trials, experience is very limited with only one trial in Los Angeles, the Field Administration of Stroke Therapy - Magnesium (FASTMAG) trial, which is currently examining the neuroprotective effect of intravenous magnesium infusion [15].

Gaps in the evidence for pre-hospital care are well recognised, and in the UK a number of national reviews are currently driving the development of infrastructure to support and enable research in this setting [14,16,17]. However, the lack of randomised controlled trials (RCT) in the pre-hospital setting is also likely to be due to greater logistical problems with their conduct. Whilst it is accepted that RCTs in any setting are challenging to perform, this is likely to be even more so in the pre-hospital domain [18,19]. Procedures such as randomisation, drug supplies and data collection are more difficult to organise with a mobile workforce operating from ambulances [18,19]. Emergency medical staff need to be trained in trial processes and then have the confidence to approach and recruit suitable patients [18,19]. In addition, emergency medical staff must be willing to participate in a trial which will usually involve duties additional to routine service provision.

Where the logistics of a randomised controlled trial are unclear, an external (rehearsal) pilot trial can be carried out to inform the design of a definitive study [20,21]. Pilot trials can be used to test protocol procedures such as the consent process, randomisation arrangements and data collection instruments. They can also be used to determine the most appropriate primary outcome measure, to estimate eligibility, recruitment and retention rates, and to inform sample size calculations for a future definitive trial [20,21].

This paper describes the protocol for a pilot randomised controlled trial of paramedic initiated lisinopril or placebo for treatment of hypertension in acute stroke. As the logistics of randomised controlled trials in the pre-hospital settling are not well established, and an acute stroke trial has particular challenges with mental capacity, communication difficulties and swallowing impairment, a pilot trial is necessary to inform the future design of a definitive pre-hospital acute stroke blood pressure lowering trial. Lisinopril has been chosen as the antihypertensive agent as it has been demonstrated to lower blood pressure after acute stroke, has a good safety profile, and can be administered sublingually. The recent Controlling Hypertension and Hypotension Immediately Post Stroke (CHHIPS) trial demonstrated the blood pressure lowering effect of lisinopril in acute stroke including the tolerability and antihypertensive effect of sublingually administered lisinopril which was given to participants with dysphagia [22]. Due to the high frequency of swallowing problems during acute stroke, the first dose of study medication is delivered sublingually in our pilot trial.

The Paramedic Initiated Lisinopril For Acute Stroke Treatment (PIL-FAST) study follows current recommendations for the design of pilot randomised controlled trials [20,21].

## Methods

### Study aim

The aim of this study is to assess the feasibility of a double blind parallel group randomised controlled trial of paramedic initiated blood pressure lowering treatment for patients with symptoms of recent stroke.

### Primary objective

To demonstrate whether it is possible to enrol at least four patients per month into the trial (from an ambulance service covering a population of 500,000).

### Secondary objectives

• To report the proportion of suspected acute stroke patients admitted to research sites during the trial duration who fulfilled the study eligibility criteria.

• To report the proportion of study eligible patients attended by a research trained paramedic.

• To report the proportion of study eligible patients enrolled into the study by a research trained paramedic.

• To report the proportion of study eligible patients approached about the research study but not enrolled, and to report reasons for non-enrolment where possible.

• To report the proportion of study eligible patients not approached about the research study, and to report reasons for non-approach where possible.

• To determine the additional time spent on scene by research trained paramedics to enrol a participant into the study.

• To report paramedic compliance with study data collection.

• To report hospital staff compliance with study medication administration and data collection.

• To report the proportion of study participants with confirmed stroke who complete seven days of study medication.

• To collect and report completeness and summary statistics on clinical data to inform the design of a definitive multicentre randomised controlled trial:

- change in blood pressure in intervention and control groups for 7 days post stroke

- change in neurological score (National Institute of Health Stroke Scale [23]) in intervention and control groups at 3 and 7 days post stroke

- dependency score (Barthel Activities of Daily Living (ADL) Index [24], Modified Rankin Scale [25]) in intervention and control groups at 7 days post stroke

- change in renal function in intervention and control groups at 7 days post stroke

- mortality in intervention and control groups at 7 days post stroke

• To report adverse events in control and intervention groups during the study.

### Study design

**Figure 1:**
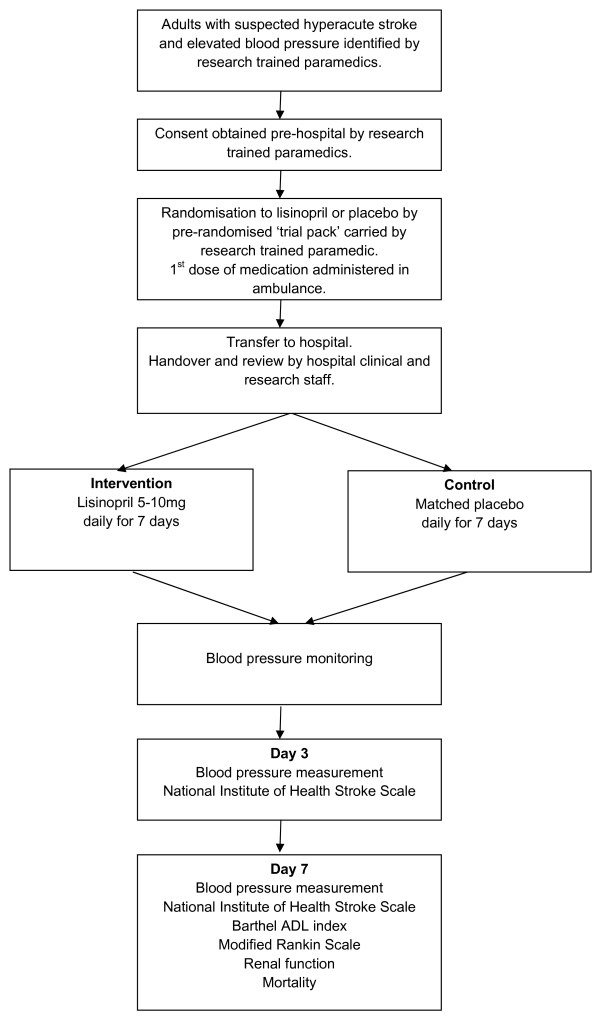
**Study method**.

The study is a double blind parallel group randomised controlled external (rehearsal) pilot trial assessing the feasibility of paramedic initiated lisinopril for acute stroke. Figure 1 outlines the study method.

### Study setting

The study is being undertaken by the North East Ambulance Service NHS Trust (NEAS), Newcastle upon Tyne Hospitals NHS Foundation Trust (Royal Victoria Infirmary) and Northumbria Healthcare NHS Foundation Trust (two sites, North Tyneside General Hospital and Wansbeck General Hospital). The study is initiated in the community by NEAS research trained paramedics, including administration of the first dose of study medication. The NEAS workforce is divided into geographical regions and the study is active in the North of Tyne division where patients are transported to the Newcastle or Northumbria hospitals which are taking part in the pilot trial. The North of Tyne division covers a population of approximately 500,000 and admits around 1200 patients with suspected stroke per year.

### Study population

Adults with suspected acute stroke presenting to NEAS North of Tyne research trained paramedics who fulfil the following criteria are eligible:

#### Inclusion criteria

• Adults ≥ 40 years old

• New unilateral arm weakness thought to be due to acute stroke within 3 hours of symptom onset

• Hypertension as defined by systolic BP >160 mm Hg on two consecutive seated or lying readings taken 5 - 10 minutes apart

• Conscious (eyes open spontaneously i.e. "A" on Alert, Voice, Pain, Unresponsive (AVPU) scale)

• Patient being transported to a PIL-FAST trial site (i.e. Royal Victoria Infirmary, North Tyneside General Hospital or Wansbeck General Hospital)

• Verbal consent obtained from participant or next of kin

#### Exclusion criteria

• Age < 40 years

• Females who are pregnant, lactating or at risk of pregnancy (i.e. who are not surgically sterile or at least 1 year post last menstrual period). Females < 56 years of age consented by a relative will be excluded as menstrual history may be unknown.

• Any presentation of suspected stroke without unilateral arm weakness

• Cannot establish that stroke onset time (i.e. when patient was last seen well without symptoms) was within the last 3 hours

• Systolic BP < 160 mm Hg

• Reduced level of consciousness (below "A" on AVPU scale)

• Patient not being transported to PIL-FAST trial site

• Absence of participant or next of kin consent

• Known to be taking Angiotensin Converting Enzyme (ACE) inhibitor or Angiotensin II Receptor Blocker medication already

• Known sensitivity to lisinopril or other ACE-inhibitor medication

• Pulse > 120 beats per minute

• Seizure activity in this illness episode (witnessed or history)

• Hypoglycaemia (blood glucose < 3.5 mmols/l)

• Cannot walk independently prior to stroke (walking stick / frame is allowed)

• Obvious understanding or memory problems when next of kin is absent

• Significant head trauma or brain surgery in the last 3 months

• Known renal failure

• Known liver failure (or currently jaundiced)

• Uncontrolled heart failure (breathlessness at rest)

• Receiving palliative care for known malignancy

• Currently enrolled in a clinical trial assessing a study drug

### Case ascertainment

Potential participants are identified by research trained paramedics using routinely collected clinical data.

### Screening, recruitment and consent

Assessment of study eligibility, invitation to participate in the study and consent is undertaken in the pre-hospital setting by research trained paramedics. Confirmation of suitability to continue study medication and provision of more detailed information is performed by the hospital stroke research team within 18 hours of admission to hospital.

#### Assessment and consent in the pre-hospital setting

The routine clinical assessment of patients with acute stroke by paramedics (i.e. symptoms and signs of stroke, physiological observations, previous medical history, current medications and drug sensitivities) provides the information needed to determine whether a patient fulfils the study inclusion and exclusion criteria. Confirmation of fulfilment of the eligibility criteria is recorded on the study case record form (CRF) following consent to enter the study.

If a potential participant meets the inclusion criteria, a simple explanation of the study is provided verbally by the attending research trained paramedic as follows:

• Your symptoms suggest you have had a stroke.

• Your blood pressure is high.

• We are working with doctors at Newcastle University to find out if it is possible to use a blood pressure lowering treatment before patients reach hospital.

• In some people this might improve recovery after stroke, but it is not proven.

• Would you be willing to take part in a study to help?

• This means receiving a small dose of a blood pressure lowering tablet or an identical "dummy" tablet before reaching hospital. The type of tablet people receive is decided by chance.

• You will be offered one or two of these extra tablets for the next seven days.

• If you agree to take part then you will be told more about the study at hospital and given the option to pull out if you change your mind.

• Your treatment and care will not be affected if you decide not to take part.

Following this explanation the research trained paramedic assesses the understanding of the potential participant by asking three questions as below.

Can I check you understood what I said about the study?

• What do we think is wrong with you at the moment? [answer: stroke]

• What did I say about your blood pressure? [answer: high]

• What will you receive if you do help with the study? [answer: tablet]

An opportunity to ask questions is then provided. The research paramedic asks "Do you have any questions about the study?" The patient is then asked to provide verbal confirmation that they are happy to take part. The consent process takes approximately three to five minutes. A longer consent process would delay transfer to hospital. Verbal confirmation of consent is recorded on the paramedic signed CRF. Following verbal consent, the patient enters the study and receives the first dose of study medication.

If a potential participant meets the inclusion criteria but does not appear to have mental capacity during the consent process or during the routine clinical assessment, it is possible for the next of kin or other close relative present at the time to act as a personal legal representative and provide consent on the basis of their understanding of the wishes of the patient. Verbal consent from the relative is recorded on the CRF.

If the paramedic is uncertain whether the patient meets the inclusion/exclusion criteria, or whether they have demonstrated sufficient understanding of the trial during the first stage of consent, they are able to contact the stroke physician on call for the locality for advice by mobile phone.

#### Provision of further study information and confirmation of appropriateness to continue study medication in the hospital setting

A more detailed study explanation and a patient information sheet is provided to the participant and/or next of kin/close relative by a member of the hospital stroke research team within 18 hours of admission to hospital. Confirmation of consent is obtained in writing from either the participant or next of kin/close relative by the admitting stroke physician. If the participant has mental capacity but is unable to sign the consent form (e.g. because of weakness of the dominant hand following stroke), consent may be confirmed orally in the presence of an independent witness (an individual not otherwise involved in the trial) who will sign the consent form on behalf of the participant. The participant (or next of kin/close relative) is free to withdraw from the study if unhappy to continue following this more detailed explanation. All participants who withdraw from the trial after receiving study medication are monitored for adverse events for seven days.

Following the availability of further clinical data, suitability to continue study medication is reviewed within 18 hours of admission by the hospital stroke research team. The following reasons make it inappropriate to continue with study medication:

• Diagnosis is found NOT to be stroke

• Revised stroke onset time was not within 3 hours of presentation to paramedics

• Female found to be at risk of pregnancy

• Documented to be taking ACE inhibitor or Angiotensin II receptor blocker

• Documented to be sensitive to lisinopril or other ACE-inhibitor medication

• Found to be in renal failure (Cr > 200)

• Found to have liver failure or to be jaundiced

• Found to have uncontrolled heart failure

• Found to be receiving palliative care for malignancy

• Found to have significant head trauma or had brain surgery in the last 3 months

• Found to be already enrolled in a clinical trial assessing a study drug

Reasons for discontinuation of study medication are recorded on the CRF. All participants continue to be followed up for the duration of the study. It is possible that one or more of the above reasons for discontinuation of study medication will only become available after 18 hours; in this event, study medication will be discontinued at this later time.

#### Design of recruitment and consent

The processes for recruitment and consent as described have been designed to enable this research to take place in the emergency setting. The consent process in emergency research should not interfere with clinical care (i.e. in this case lead to a lengthy delay in transfer to an acute stroke unit) but should "take due account of the views, however expressed, by the person being treated or by their family or friends who are with them" [26]. If a traditional consent process is not undertaken at the time of the emergency, the recommendation is that: "as soon as the emergency is over, arrangements must be made to seek consent in the usual manner, or to seek advice from a consultee on the continued participation of the person who lacks capacity in the study" [26].

As stroke can create impairments which interfere with communication and mental capacity, the consent process incorporates a simple capacity assessment and the views of relatives will be sought about trial participation where mental capacity is impaired. Relative consent has been used in other trials of emergency neurological conditions (Corticosteroid Randomisation After Significant Head Injury (CRASH) trial which examined the role of steroids in the emergency treatment of acute head injury [27] and the National Institute of Neurological Disorders and Stroke (NINDS) trial which evaluated intravenous thrombolysis for stroke [28]). Relative consent is also used in current randomised controlled trials of emergency stroke treatments including the 3^rd ^International Stroke Trial [29] and Stroke Oxygen Study [30]. We believe the design of our consent process is unique as we have developed a standardised approach for non medically qualified ambulance paramedics to assess understanding of the trial to support consent in the pre-hospital setting. Exclusion of patients with communication or mental incapacity would mean exclusion of patients with more disabling strokes who may have the most to gain from treatment.

### Study medication

Participants receive lisinopril (5-10 mg) or matched placebo daily for 7 days. Lisinopril is a licenced medication for the treatment of hypertension but by initiation within the hyperacute phase of stroke it is used outside of the specific indications in the current licence and is being treated as an investigational medicinal product (IMP) in this trial.

Lisinopril and matched placebo tablets are blister packaged and boxed (16 tablets per box). Boxes are identical and each carries a unique study number linked to the randomisation code. Modepharma, a clinical trial supplies company, supplied the boxed lisinopril and matched placebo which were obtained from Haupt Pharma Wuelfing (Germany).

The first participant treatment is 5 mg (1 tablet) administered by a research trained paramedic following study entry. As lisinopril (and placebo) is not a routine pre-hospital drug, a trial specific Patient Group Directive (PGD) was developed and subsequently approved by the North East Ambulance Service NHS Trust to enable research trained paramedics to administer trial medication. Due to the high frequency of swallowing problems during acute stroke, the first dose is delivered sublingually. The 5 mg tablet (lisinopril or matched placebo) is crushed in a syringe crusher with 3 - 5 mls of water and then administered under the tongue. The assessment of swallowing safety cannot be done by paramedics and swallowing safety may change rapidly.

Following arrival of a participant at hospital, review of the appropriateness to continue study medication and provision of further information (as above), the stroke physician makes a decision about dose titration. Three blood pressure readings are taken and if the systolic blood pressure (SBP) remains > 150 mmHg, a second dose of lisinopril or matched placebo (5 mg) is administered. Route of administration is either oral, sublingual or via nasogastric tube, dependent on swallow safety, which is assessed as part of routine care after admission. If the SBP is < 150 mmHg a second dose is NOT administered. A daily dose of 5 mg (one tablet) is given if dose escalation has not been necessary and 10 mg (two tablets, as a single daily dose) if dose escalation was performed. Route of administration is either oral, sublingual or via nasogastric tube, dependent on swallow safety which may change over the course of the study.

If already prescribed pre-stroke, participants continue on their usual blood pressure lowering medication as well as the trial medication. Without thrombolysis, if systolic blood pressure remains below 200 mmHg during the first 7 days then no additional blood pressure lowering medication will be introduced until after the day 7 assessment, unless the clinical team judge it is important for the blood pressure to be lowered more urgently according to the condition of the patient. If the systolic blood pressure exceeds 200 mmHg within the trial intervention period, the clinical team will decide on additional blood pressure lowering treatment. If patients in the trial receive thrombolysis and their blood pressure rises above 185 mmHg then clinical treatment protocols dictate that additional medication will be started to reduce blood pressure below this level for 24 hours, after which clinical judgement will determine whether additional new blood pressure lowering measures are required in addition to the trial medication.

If a participant develops clinically significant hypotension (i.e. symptomatic < 120 mmHg), trial medication will be withheld and all pre-trial blood pressure lowering medications being administered will be reviewed. If blood pressure increases to >150 mmHg trial drugs will be reintroduced at the 5 mg dose. All medication changes are noted on the CRF. Daily dosage of study medication and compliance with treatment is also recorded on the CRF.

If a participant is discharged from hospital before seven days, they will take the remaining tablets at home. Following completion of the study, the stroke physician will decide on any further appropriate blood pressure lowering treatment required by the patient on the basis of clinical assessment.

### The PIL-FAST 'trial pack'

In order to enable access to study medication and the necessary administration equipment in the pre-hospital setting, boxes of lisinopril and matched placebo, along with other study materials, have been secondarily packaged into a 'trial pack'. The trial pack contains the paramedic research paperwork, study medication, a syringe crusher, a 5 ml syringe and 5 ml vial of water. Each pack carries a unique study number which matches the medication box number (linked to the randomisation code). Packaging of trial packs was carried out by the Newcastle upon Tyne Hospitals Pharmacy Production Unit.

The provision of trial packs to research trained paramedics mirrors the provision of routine ambulance drug supplies to the North East Ambulance Service which are provided by Lloydspharmacy, a community pharmacy, in weekly deliveries to ambulance stations. Once packaged by the Newcastle upon Tyne Hospitals Pharmacy Production Unit, the trial packs were transferred to the local Lloydspharmacy in Gateshead who are storing and delivering the packs to participating North of Tyne ambulance stations. Lloydspharmacy delivery staff place packs in the ambulance station drug rooms and replenish to meet a minimum stock supply on their weekly visit to restock routine drug supplies.

A trial pack is collected by each research trained paramedic, taken into their ambulance service vehicle and carried on their emergency shift. If the pack is unused at the end of the shift, it is stored in the paramedic's individual secure locker until their next shift. This approach is being employed due to the logistics of drug storage on ambulance service vehicles and because the vehicles will not always carry a research trained paramedic. Second and subsequent packs are collected once the first pack has been used. Packs are opened after an eligible patient gives consent to take part in the trial and contents accompany the participant to hospital.

In addition to the contents listed above, 20% of the trial packs also contain a small temperature monitoring device. This is because ambulance service vehicles do not contain temperature controlled storage spaces and trial medication should not be stored above 25°C. Temperature monitoring is also being performed in a sample of ambulance station storage areas which similarly are not routinely temperature controlled.

### Paramedic training

Paramedics taking part in the study are volunteers from the NEAS North of Tyne division. In order to participate, they must have attended a training day which covered the principles of Good Clinical Practice (GCP), stroke recognition and trial eligibility assessment, the trial consent process and other trial procedures. In addition, all research trained paramedics have been issued with a small laminated booklet, designed to be carried on emergency shifts, which details inclusion and exclusion criteria, the consent script and emergency contact numbers.

### Randomisation

Treatment allocation is according to the trial pack carried by the recruiting paramedic. The trial packs carry unique study numbers identical to the numbers on the boxes of lisinopril and matched placebo contained within. The numbers are according to a master randomisation list created by an independent statistician. Intervention and control allocations are in 1:1 ratio.

### Blinding

Participants, clinicians (paramedics, investigators, outcome assessors) and other trial staff are blinded to treatment allocation. Lisinopril and placebo tablets are identical in appearance and packing. In the event of an emergency (as confirmed by the local site investigator, chief investigator or emergency doctor if investigators unavailable), the on-call pharmacist at the trial pharmacy can be contacted to reveal treatment allocation.

The integrity of blinding is checked at the final participant assessment. The participant is asked if they believe they received lisinopril or placebo. The assessor is also asked to record the treatment that they believe the participant to have received.

### Baseline data

In addition to the baseline eligibility data collected by research trained paramedics, the following are collected from routine records by a member of the hospital stroke research team following arrival of the participant at hospital: place of residence, past medical history, medication prior to admission, stroke type, brain imaging result, electrocardiogram result (rate, rhythm, voltage criteria for left ventricular hypertrophy (LVH)), renal function (urea and creatinine) on admission.

The following assessments are also performed within 18 hours of admission to hospital:

• Pre-stroke Barthel ADL Index score [24]

• Pre-stroke Modified Rankin Scale score [25]

• National Institute of Health Stroke Scale score [23]

Furthermore, at the hospitals taking part in this research study, the stroke research teams routinely screen the records of all patients admitted with a diagnosis of suspected acute stroke. Data from this screen is being used to identify all patients who fulfilled the study eligibility criteria and those that were transported to hospital by a research trained paramedic. For patients transported to hospital by a research trained paramedic and study eligible but not enrolled, paramedic routine clinical records are examined and any details about study screening or approach recorded if available.

In order to assess the additional time spent on scene by paramedics enrolling trial participants, time from arrival on scene to hospital door is obtained for all stroke admissions to the research hospitals from NEAS data records.

### Outcome assessments

Outcome data is collected from (i) routine clinical records and (ii) specific research assessments, by a member of the hospital stroke research team.

(i) Blood pressure during the first 48 hours following study entry is transcribed from routine clinical records onto the study CRF. Blood pressure at hospital admission, and at 4, 24 and 48 hours after admission is recorded (blood pressure is also recorded at the study medication dosage review, as above).

(iii) Specific research assessments take place at (a) day 3 after study entry and (b) day 7 (+/- 1 day) after study entry.

(a) On day 3 after study entry the following assessments are performed:

• Blood pressure (3 readings)

• National Institute of Health Stroke Scale

(b) On day 7 (+/- 1 day) after study entry the following assessments are performed:

• Blood pressure (3 readings)

• National Institute of Health Stroke Scale

• Barthel ADL Index

• Modified Rankin Scale

• Blood test for renal function, (urea, creatinine)

If a participant is discharged before day 3 or 7 following study entry, they will be asked to attend the outpatient department for data collection.

The study schedule is shown in Table 1.

**Table 1:** Study schedule

	Day 1	Day 2	Day 3	Day 4	Day 5	Day 6	Day 7
Eligibility assessed by paramedic from routine clinical data	x						

Study discussed and verbal informed consent/assent obtained by paramedic	x						

Randomisation	x						

Further study information provided and written informed consent/assent obtained by hospital research team	x						

Appropriateness to continue study medication reviewed by hospital team	x						

Review of medical records to collect baseline stroke details and medical history	X						

Blood pressure measurements	x	x	x				x

National Institute of Health Stroke Scale	x		x				x

Barthel ADL Index (pre-stroke)	x						

Barthel ADL Index (post-stroke)							x

Modified Rankin Scale (pre-stroke)	x						

Modified Rankin Scale (post-stroke)							x

Blood test for renal function							x

Review of medical records to record concomitant blood pressure medications							x

Treatment with study medication	x	x	x	x	x	x	x

Review of study medication dosage	x						

Adverse Events	x	x	x	x	x	x	x

### Study withdrawal

No specific study withdrawal criteria have been pre-set. Participants may withdraw from the study at any time for any reason. Should a patient decide to withdraw from the study, a reason will be sought (to allow the study team to assess likely barriers to participation and retention) but patients can chose to withdraw without providing an explanation. Participants who chose to withdraw are monitored for adverse events for seven days after initiation of study medication. Patients wishing to withdraw prematurely from study medication are asked if they would be willing to continue with follow-up assessments as per the study schedule.

Investigators may also withdraw participants from the study at any time if they feel it is no longer in their interest to continue, for example, because of intercurrent illness or adverse events. Withdrawn participants are not replaced.

### Pharmacovigilance

The safety of lisinopril 5-10 mg in acute stroke will be evaluated by examining the occurrence of all adverse events as defined by the Medicines for Human Use (Clinical Trials) Regulations [31].

For serious adverse events (SAE), relationship to treatment (causality) and expectedness is determined by the local site investigator. Any queries will be discussed with the Chief Investigator. Excepting common and anticipated stroke complications (pneumonia, urinary tract infection, seizure, headache, deep vein thrombosis, pulmonary embolism), all SAEs will be reported to the co-ordinating centre immediately (within 24 hours) to comply with the Medicines for Human Use (Clinical Trials) Regulations [31]. Anticipated stroke complications need not be reported within 24 hours.

The Medicines and Healthcare products Regulatory Agency (MHRA) and ethics committee will be notified of all suspected unexpected serious adverse reactions (SUSAR) according to the following timelines in line with the Medicines for Human Use (Clinical Trials) Regulations: fatal and life threatening within 7 days of notification, non life threatening within 15 days. SUSARs will also be notified to all trial sites.

For non serious adverse events, local site investigators will determine relationship to treatment and data will be processed with routine trial data.

#### Pregnancies

If a pregnancy is confirmed in a female participant during her participation in the trial, the study drug will be discontinued immediately. The pregnancy will be followed up to determine the outcome. Additional follow-up will no longer be required once the newborn is determined to be healthy.

### Statistical analysis

As this is a pilot study, the statistical analysis will be descriptive in nature, providing estimates of key trial parameters and to inform power calculations for a future definitive trial. Formal statistical comparisons between randomisation groups will not be undertaken.

#### Sample size calculation

As this is a pilot study, a formal sample size calculation has not been carried out. We aim to recruit and randomise 60 participants; collecting data on 30 patients per randomisation group is acceptable for calculating sample sizes [20]. We believe that approximately 200 patients per year who fulfil the study eligibility criteria will present to NEAS within the research boundaries.

### Ethical arrangements and research governance

The study is being conducted in accordance with the principles of Good Clinical Practice (GCP) [32], the Medicines for Human Use (Clinical Trials) Regulations [31] and the Research Governance Framework for Health and Social Care [33]. Ethical, NHS Trust and MHRA approvals have been obtained. The study sponsor is Newcastle upon Tyne Hospitals NHS Foundation Trust.

A Data Monitoring and Ethics Committee (DMEC) and Trial Steering Committee (TSC) have been convened.

### Current study status

The study commenced on 29^th ^October 2010. Seventy six paramedics have received PIL-FAST training. Recruitment will be until end of December 2011. Results will be submitted for publication in 2012.

## Discussion

Systemic hypertension in acute stroke is associated with poorer stroke outcome [1,2], but optimal treatment of hypertension early after stroke is unclear [6]. Previous randomised controlled trials of blood pressure lowering agents may have failed to show improved functional outcome due to treatment being administered too late after stroke to be effective. The earliest time after stroke that treatment could be initiated is during contact with the emergency medical services (paramedics).

This paper describes a protocol for a pilot randomised controlled trial of paramedic initiated lisinopril for hypertension in acute stroke. Pilot RCTs are important to perform when the logistics of a large scale trial are unclear [20,21]. Experience of pre-hospital RCTs, especially pre-hospital stroke trials, is very limited, making a pilot study a necessary and important step of a definitive evaluation.

This pilot study will inform the design of a large multicentre randomised controlled trial to evaluate very early blood pressure lowering in acute stroke. We will determine if paramedic recruitment to an acute stroke trial is feasible along with the estimated recruitment rate that could be achieved. We are testing a novel consent process, and method for randomisation, availability of trial medication and supporting equipment (the trial pack). We will also review the completeness of paramedic and hospital staff data collection, both of which are important for a definitive study. In addition, we hope to capture any unanticipated protocol logistical issues integral to the success of a future large scale RCT.

If the feasibility of this pre-hospital acute stroke trial is demonstrated, it will allow the conduct of a definitive RCT to evaluate the effects of very early blood pressure lowering in acute stroke. It will also pave the way for evaluation of other potential paramedic initiated treatments where very early intervention may improve outcome.

## List of abbreviations

ACE: Angiotensin Converting Enzyme; ADL: Activities of Daily Living; AVPU: Alert, Voice, Pain, Unresponsive; CHHIPS: Controlling Hypertension and Hypotension Immediately Post Stroke; CRASH: Corticosteroid Randomisation After Significant Head Injury; CRF: Case Record Form; DMEC: Data Monitoring and Ethics Committee; FASTMAG: Field Administration of Stroke Therapy - Magnesium; GCP: Good Clinical Practice; IMP: Investigational Medicinal Product; LVH: Left Ventricular Hypertrophy; MHRA: Medicines and Healthcare products Regulatory Agency; NEAS: North East Ambulance Service NHS Trust; NIHR: National Institute of Health Research; NINDS: National Institute of Neurological Disorders and Stroke; PGD: Patient Group Directive; PIL-FAST: Paramedic Initiated Lisinopril For Acute Stroke Treatment; RCT: Randomised Controlled Trial; SAE: Serious Adverse Event; SBP: Systolic Blood Pressure; SUSAR: Suspected Unexpected Serious Adverse Reaction; TSC: Trial Steering Committee

## Competing interests

The authors declare that they have no competing interests.

## Authors' contributions

LS wrote the study protocol, created the CRFs, orchestrated drug supplies, study approvals and site set up, and drafted this paper. CP and GAF designed the trial and had input into all study documents, processes and site set up. GAF is the chief investigator. SM contributed to study design and orchestrated the input of NEAS. DH contributed to the study design and is the study statistician. EM contributed to study design and supervised the involvement of Newcastle Clinical Trials Unit.

All authors have commented upon drafts of the paper and have given final approval to this version.
